# Tumor Bed Radiosurgery Following Resection and Prior Stereotactic Radiosurgery for Locally Persistent Brain Metastasis

**DOI:** 10.3389/fonc.2015.00084

**Published:** 2015-04-08

**Authors:** Douglas Emerson Holt, Beant Singh Gill, David Anthony Clump, Jonathan E. Leeman, Steven A. Burton, Nduka M. Amankulor, Johnathan Anderson Engh, Dwight E. Heron

**Affiliations:** ^1^Department of Radiation Oncology, University of Pittsburgh Cancer Institute, Pittsburgh, PA, USA; ^2^Department of Radiation Oncology, Memorial Sloan Kettering Cancer Center, New York, NY, USA; ^3^Department of Neurological Surgery, University of Pittsburgh Medical Center, Pittsburgh, PA, USA

**Keywords:** radiosurgery, brain metastases, re-irradiation, recurrence, cyberknife

## Abstract

**Purpose:**

Despite advances in multimodality management of brain metastases, local progression following stereotactic radiosurgery (SRS) can occur. Often, surgical resection is favored, as it frequently provides immediate symptom relief as well as pathological characterization of any residual tumor. Should the pathological specimen contain viable tumor cells, further radiation therapy is an option to sterilize the tumor bed. We evaluated the use of repeat SRS (rSRS) in lieu of whole-brain radiation therapy (WBRT) as a means of improving local control (LC) while minimizing potential toxicity and dose to the normal brain.

**Materials/methods:**

A retrospective review was performed to identify patients with brain metastases who underwent SRS and then surgical resection for locally recurrent or persistent disease. From 2004 to 2014, 13 consecutive patients or 15 lesions were treated with rSRS after resection, either post-operatively to the tumor bed (*n* = 10, 66.6%) or after a second local recurrence (*n* = 5, 33.3%). LC, distant brain failure (DBF), and radiation toxicity were determined using patient records, RECIST criteria v1.1, and CTCAE v4.03.

**Results:**

At a median follow-up interval of 9.0 months (range 1.8–54.9 months) from time of rSRS, five patients remain alive. Following rSRS, 13 of the 15 (86.6%) lesions were locally controlled with an estimated 100% LC at 6 months and 75% LC at 1 year. However, 11 of the 15 (73.3%) treated lesions developed DBF after rSRS with 3 of 13 patients proceeding to WBRT. Two of 15 (13.3%) resulted in either grade 2 radionecrosis with grade 3 seizures or grade 3 radionecrosis.

**Conclusion:**

Repeat SRS represents a potential salvage therapy for patients with locally recurrent brain metastases, providing additional tumor control with acceptable toxicity, even in the setting of prior SRS and surgical resection. rSRS may be reasonable to use as an alternative to WBRT in this setting.

## Introduction

Metastatic brain disease is a frequent cause of morbidity and mortality in patients with cancer, occurring at rates as high as 40% (1–3). Without treatment, the prognosis is often poor, with survival usually limited from weeks to months, frequently from neurological death (4). The mainstays of treatment for brain metastases include whole-brain radiotherapy (WBRT), stereotactic radiosurgery (SRS), and surgical resection. WBRT has been the primary treatment of brain metastases; however, it has been associated with neurocognitive decline and decreased quality of life (5, 6). Definitive SRS has benefits of excellent reported local control (LC) rates, minimal invasiveness, and low risks of radiation toxicity (7). Surgical resection may be indicated if the lesion is large, progressive, and/or hemorrhagic causing a mass effect. If resection is sought, it is usually combined with adjuvant radiotherapy due to high local recurrence rates associated with surgery alone (8).

As patients continue to live longer with metastatic brain disease, local brain relapse and distant brain failure (DBF) may occur more frequently, thus necessitating the treatment and management of recurrent brain metastases. Salvage therapy options include repeat SRS (rSRS), surgery, and WBRT. Unfortunately, there are no randomized clinical trials for the retreatment of recurrent brain metastatic disease. Nonetheless, there are a limited number of studies and reports discussing salvage treatments; thus, their utility and use may be extrapolated from the observational studies along with clinical judgment. Therefore, the treatment plans are often individualized, depending on many factors such as prior therapy, size, location, number of lesions, performance status, status of systemic disease, symptoms, and graded prognostic assessment (9). There have been concerns with tissue tolerance with re-irradiation (10, 11). However, neurological complications from rSRS have been reported to be minimal (12). Furthermore, acceptable dose ranges of SRS were observed for previously irradiated brain tumors with a range 15–24 Gy depending on tumor size (13).

In this unique case series, we present the clinical outcomes of patients who had metastatic brain lesions initially treated with definitive SRS, followed by surgical resection and rSRS for recurrent brain disease.

## Materials and Methods

Following Institutional Review Board approval (PRO 13020306), a retrospective review of all patients treated with SRS for metastatic brain disease was completed. Patients were treated between September 2004 and May 2014 at the University of Pittsburgh Cancer Institute, initially consisting of 1189 patients. Thirteen patients (15 lesions) were identified who successfully completed the treatment regimen sequence of SRS, surgical resection, and rSRS to the same or adjacent location. Surgical resection was done following initial SRS due to either locally recurrent or persistent disease. The definition of the adjacent location was based on a close proximity to the previously irradiated site, such that the rSRS treatment field would overlap with the previously treated field. Pre-treatment data and patient characteristics collected included diagnosis, tumor location, interval between treatments, treatment volumes and doses for each session, baseline and subsequent neurologic symptoms, and radiographic evidence of change in tumor size. Initial SRS doses were delivered according to treatment volume; however, for rSRS re-irradiation of previously resected lesions, delivered dose was often fractionated to possibly reduce radiation-related toxicities. Systemic therapies were not evaluated in this patient population due to incomplete records for this treatment modality.

Local failure (LF) and DBF were determined based on symptomatic and radiographic progression, utilizing the Response Evaluation Criteria in Solid Tumors version 1.1 (RECIST v1.1) (14). Treatment-related toxicities such as radionecrosis and seizures were scored using Common Terminology Criteria for Adverse Events version 4.03 (CTCAE v4.03). An increase in any of the neurological symptoms or new symptoms after re-irradiation without disease progression was considered radiation treatment effect. Survival, LC, and DBF were estimated using the Kaplan–Meier method from the time of either SRS or rSRS to the data of failure or last follow-up/death. Statistical significance was defined with a critical value of *p* < 0.05. Kaplan–Meier analysis and univariate Cox proportional hazards regression with frailty model for correlated data were used with Stata version 13 (15).

## Results

Baseline patient characteristics are outlined in Table 1. Briefly, the study population consisted of 13 patients (15 treatments) with a median age of 54 years who underwent rSRS to a tumor cavity after initial SRS treatment and surgical resection. The most common tumor histologies were melanoma (60%) and breast (13.3%) cancers. One patient had received prior WBRT before initial SRS. After initial SRS, surgery was sought due to tumor progression and/or hemorrhagic mass effect. The intent of treatment of rSRS to the tumor bed was for adjuvant therapy with resection in 10 of the 15 lesions (66.7%), whereas the other 5 (33.3%) were for local progression post-resection. Also, eight (61.5%) of the patients treated had no active extracranial disease at time of delivery of rSRS to the resection cavity.

**Table 1:** **Baseline patient characteristics**.

Patient characteristics	*n* = 13 patients (*n* = 15 lesions)
**Age**
Median (range)	53 years (30–70 years)
**Gender**	5 males, 8 females
**KPS**
Median (range)	80 (70–90)
**Initial GPA score**
Median (range)	2 (1–3)
**Initial RPA score**
Median (range)	2 (1–2)
**Primary histology**
Melanoma (%)	9 (60.0)
Breast (%)	2 (13.3)
Lung (%)	1 (6.7)
Renal (%)	1 (6.7)
Colon (%)	1 (6.7)
Endometrial (%)	1 (6.7)
**Radiotherapy prior to repeat SRS**
Median number of prior SRS treatments excluding repeat SRS (range)	3 (1–6)
Whole-brain radiotherapy (%)	1 (6.7)
**Number of active brain metastases at repeat SRS**
Median (range)	0 (0–4)
**Extracranial disease controlled at repeat SRS**
Yes (%)	8 (53.3)
No (%)	7 (46.7)
**Treatment intent of rSRS to tumor bed**
Adjuvant/prophylactic for local control (%)	10 (66.7%)
Control of recurrent disease (%)	5 (33.3%)

**Figure 1:**
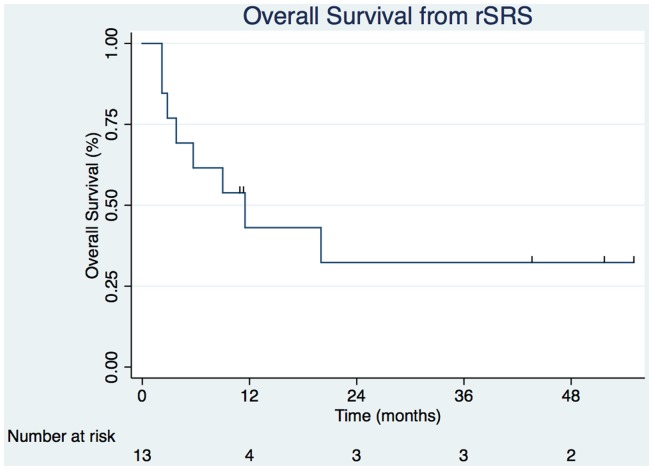
**Overall survival from rSRS (hash marks represent censored observation)**.

Table 2 displays the SRS and rSRS treatment characteristics along with clinical outcomes. The median time period from SRS to rSRS was 6.4 months (2.4–15.2 months). The overall median time from rSRS to last follow-up was 9.0 months (2.2–54.9 months). Five (38.5%) patients were alive at last follow-up. The 6- and 12-month estimates of overall survival from rSRS are 61.5% (30.8.3–81.8%) and 43.1% (8.6–59.4%), respectively (Figure 1). Patients with melanoma histology associated with an increased risk of death (*p* = 0.049, 95% CI 1.01–99.3).

**Table 2:** **SRS and rSRS characteristics and clinical outcomes**.

	SRS	rSRS
**Median dose (range)**	21 Gy (18–27 Gy)	21 Gy (16–30 Gy)
**Median volume (range)**	4.3 cc (0.76–19.3 cc)	9.4 cc (0.57–23 cc)
**Median number of fractions** (range)	1 (1–1)	3 (1–3)
**Isodose**	80%	80%
**Treatment platform**
Cyberknife	15	13
Trilogy	–	1
TrueBeam	–	1
**Median time from SRS to resection** (range)		**3.8 months** (0.5–14.2 months)
**Median time from resection to rSRS** (range)		**1.1 months** (0.7–4.4 months)
**Median time from SRS to rSRS** (range)		**6.4 months** (2.4–15.2 months)
**Overall survival from SRS**	
Median follow-up (range)		**13.3 months** (4.6–60.5)
6-month Kaplan–Meier estimate (95% CI)		**93.3%** (61.3–99.0%)
12-month Kaplan–Meier estimate (95% CI)		**53.3%** (26.3–74.4%)
**Overall survival from rSRS**		
Median follow-up (range)		**9.0 months** (2.2–54.9)
Patients alive at last follow-up (%)		**5 of 13** (34.5%)
6-month Kaplan–Meier estimate (95% CI)		**61.5%** (30.8.3–81.8%)
12-month Kaplan–Meier estimate (95% CI)		**43.8%** (8.6–59.4%)
**Local control from rSRS**	
Crude (%)		**13 of 15** (86.7%)
6-month Kaplan–Meier estimate (95% CI)		**100.0%**
12-month Kaplan–Meier estimate (95% CI)		**75.0%** (31.5–93.1%)
**Distant brain control from rSRS**	
Crude (%)		**4 of 15** (26.6%)
6-month Kaplan–Meier estimate (95% CI)		**56.6%** (27.3–77.9%)
12-month Kaplan–Meier estimate (95% CI)		**40.4%** (15.2–64.7)

**Figure 2:**
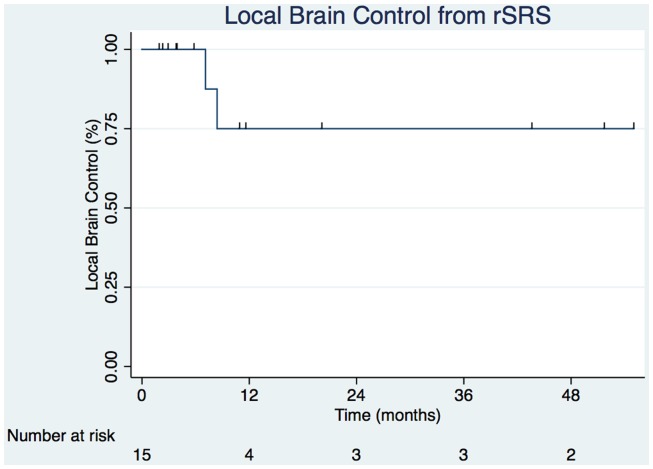
**Local control from rSRS (hash marks represent censored observation)**.

Crude LC of the tumor bed from rSRS was 86.7% with the estimated Kaplan–Meier 6- and 12-month survivals at 100 and 75.0% (31.5–93.1%), respectively (Figure 2). The crude DBF rate from rSRS was 73.3% with estimated Kaplan–Meier distant brain control rates of 56.6% (27.3–77.9%) and 40.4% (15.2–64.7) at 6- and 12-months, respectively. Of note, there were two patients who had neither LF nor DBF after rSRS, albeit with 2.2 and 2.8 months follow-up given progression of extracranial disease resulting in death.

Of the 11 patients with recurrent disease either as local or DBF after salvage SRS, 1 succumbed to rapid neurological deterioration leading to death; 2 pursued supportive care alone; 5 were treated with additional SRS, and the remaining 3 were given WBRT (Table 3).

**Table 3:** **Description of definitive treatments and outcomes**.

Age at SRS	Histology	Time from SRS to rSRS (months)	Extracranially active disease at rSRS	rSRS PTV (cc)	rSRS dose Gy (fractions)	Time rSRS to LF (months)	Time rSRS to DBF (months)	Treatment for recurrent disease	Time to Last follow-up from rSRS (alive)
29	Melanoma	4.2	No	19.0	24 (3)	–	2.3	SRS	5.7
51	Breast	15.2	No	6.3	20 (3)	7.1	–	SRS	11.2 (alive)
52	Breast	7.4	Yes	9.4	18 (1)	–	–	–	2.8
69	Melanoma	2.4	No	10.5	30 (3)	–	–	–	2.2
59^a^	Melanoma	7.3	Yes	6.6	21 (3)	–	3.5	Palliation	3.7
59^a^	Melanoma	4.5	Yes	2.4	16 (1)	–	3.5	Palliation	3.8
40	Melanoma	2.5	No	0.6	21 (1)	–	7.5	SRS	10.8 (alive)
62	Lung	9.0	No	5.4	24 (3)	–	16.4	SRS/WBRT	51.6 (alive)
52	Renal cell	4.7	Yes	9.7	18 (1)	–	17.5	SRS	54.9 (alive)
52	Melanoma	9.9	No	3.8	18 (1)	8.4	–	Palliation	9
61	Melanoma	7.8	Yes	21.0	22 (3)	–	1.1	SRS/WBRT	11.5
63	Endometrial	6.6	No	23.0	22 (3)	–	8.3	WBRT	20
52	Colon	3.7	No	9.8	24 (3)	–	20.6	SRS	43.5 (alive)
56^b^	Melanoma	6.3	Yes	4.8	18 (1)	–	0.7	–	1.8
56^b^	Melanoma	5.8	Yes	9.6	22 (3)	–	1.1	–	2.2

*^a^Same patient*.

*^b^Same patient*.

There were two patients who experienced radiation-related toxicity after rSRS. One developed radionecrosis at 1.5 months requiring steroids (grade 2) and seizures from a temporal lesion requiring multiple admissions and a complex multi-drug regimen for control (grade 3). The second patient demonstrated radionecrosis at 4.8 months post-rSRS requiring Avastin (grade 3).

## Discussion

Radiosurgery to the tumor bed following surgical resection with prior SRS appears feasible as a salvage approach in patients who have locally recurrent brain tumors. Using varying treatment platforms, doses ranging from 16 to 30 Gy in one to three fractions and a median planning treatment volume of 9.4 cc, the demonstrated 1-year local progression-free survival is 75% and overall median survival of 11.2 months from rSRS with 13.3 months from initial SRS. At the present time, there are no known studies of this treatment paradigm. However, there are two smaller known case series that evaluated rSRS for LF previously treated with SRS. Jayachandran et al. and Minniti et al. reported median OS of 26 and 10.3 months, respectively (16, 17). Moreover, there are five known cases series presenting the clinical outcomes of rSRS for recurrent distant brain metastatic disease after prior SRS, with only three of the studies presenting overall survival from time of rSRS. Chen et al., Kwon et al., and Mariya et al. presented median survivals from rSRS of 6.5, 7.3, and 11 months, respectively (18–20). Though the median survival reported from all of the studies from initial SRS was somewhat broader in range of 11.5–26 months (18–22). In comparison, our clinical outcomes are comparable to these prior published results for rSRS treatments, with the additional treatment of surgical resection.

Repeat SRS has the ability to precisely target an intracranial lesion or cavity with high dose irradiation while limiting exposure to surrounding normal tissue. Nonetheless, re-irradiation particularly after radiosurgery has been cautiously approached due to concerns for radionecrosis. The re-irradiation toxicity rates are limited in the literature for previous rSRS studies. Kwon et al. reported rates of symptomatic radionecrosis of 18.6% though did not distinguish between rSRS for locally recurrent and DBF (18, 19). Bhatnagar et al., who investigated the use rSRS in primary and metastatic brain lesions, reported an overall radionecrosis rate of 11.5% identified by MRI, although these patients were asymptomatic (12). Recently, Jayachandran et al. reported radiation-related toxicity rates of 14.8% (4 of 27 lesions) (16). In the present series, severe toxicity rates were acceptable at a crude rate of 13.3%, with 84.6% of the patients able to complete the prescribed retreatment course without interruption or complication. Resection between SRS treatments may have in fact aided in limiting radiation toxicity, since the previously irradiated tissue was removed, thus having a lower amount of tissue being re-irradiated at a high dose. However, the larger treatment volume of tumor bed SRS may have increased the risk of radiation-related toxicities. Regarding this care series, salvage SRS was often fractionated (one to three fractions) to potentially reduce radiation-related toxicities in the setting of re-irradiation. Perhaps, a higher fraction schedule (three to five fractions) may be more appropriate to limit the risk of toxicity with re-irradiation by reducing the biologically effective dose. There are now a number of studies that have evaluated upfront treatment of hypofractionated SRS for primarily large brain metastases and have reported reasonable rates of adverse events with favorable LC for both intact (23–25) and resected lesions (26).

In the context of alternative treatment options, these rates of toxicity may be more acceptable than the potential neurocognitive decline and reduced quality of life seen especially with long-term survivors of WBRT (5, 6, 27). Additionally, current literature suggests that systemic therapy has been largely ineffective in the management of most brain metastases, primarily due to poor blood–brain barrier penetrability, sub-therapeutic drug concentrations in the periphery of lesions (28), and chemo-resistivity (29, 30). In select groups and with newer biological agents, improved blood–brain barrier penetration may lead to improvements in intracranial disease control (31–34).

Of note, patients with histologically positive melanoma were associated with increased mortality (*p* = 0.049), despite the small sample size of this study. The median survival for melanoma was 3.8 months compared to the overall median of 11.2 months. This effect on survival is likely due to the aggressive nature of melanoma with a high propensity for DBF and extracranial progression (35). As a result, judicious patient selection should be used in this population with a limited life expectancy.

Given the relatively uncommon incidence of this treatment course, this case series is limited by its small sample size and patient selection bias, which should be taken into consideration when reviewing feasibility and safety. However, the presented data are unique and should re-assure oncologists that properly selected patients may benefit from this salvage approach given limited toxicity from re-irradiation. Similarly, repeat radiosurgery following surgical resection can provide satisfactory rates of LC in lieu of WBRT. Further prospective studies should ultimately evaluate the role of rSRS for patients with recurrent brain metastases in appropriately selected patients.

## Conclusion

Stereotactic radiosurgery after surgical resection and prior radiosurgery appears to be feasible with a rare risk of late toxicity, namely radionecrosis. This approach allows withholding of WBRT to potentially avoid neurocognitive deficits earlier in the patient’s course. However, these patients are at substantial risk for developing DBF and thus should be managed with close imaging surveillance.

## Conflict of Interest Statement

The authors declare that the research was conducted in the absence of any commercial or financial relationships that could be construed as a potential conflict of interest.

## Author Note

Data were presented in abstract form at The Radiosurgery Society Symposium (Minneapolis, MN, USA, May 2014).
